# Unbalanced Metalloproteinase-9 and Tissue Inhibitors of Metalloproteinases Ratios Predict Hemorrhagic Transformation of Lesion in Ischemic Stroke Patients Treated with Thrombolysis: Results from the MAGIC Study

**DOI:** 10.3389/fneur.2015.00121

**Published:** 2015-05-27

**Authors:** Benedetta Piccardi, Vanessa Palumbo, Mascia Nesi, Patrizia Nencini, Anna Maria Gori, Betti Giusti, Giovanni Pracucci, Paolina Tonelli, Eleonora Innocenti, Alice Sereni, Elena Sticchi, Danilo Toni, Paolo Bovi, Mario Guidotti, Maria Rosaria Tola, Domenico Consoli, Giuseppe Micieli, Rossana Tassi, Giovanni Orlandi, Francesco Perini, Norina Marcello, Antonia Nucera, Francesca Massaro, Maria Luisa DeLodovici, Giorgio Bono, Maria Sessa, Rosanna Abbate, Domenico Inzitari

**Affiliations:** ^1^Neuroscience Section, Department of Neurofarba, University of Florence, Florence, Italy; ^2^Stroke Unit, Department of Neurology, Careggi University Hospital, Florence, Italy; ^3^Department of Experimental and Clinical Medicine, Atherothrombotic Diseases Center, AOU Careggi, University of Florence, Florence, Italy; ^4^Emergency Department Stroke Unit, Department of Neurological Sciences, Sapienza University of Rome, Rome, Italy; ^5^SSO Stroke Unit, U.O. Neurologia d.O., DAI di Neuroscienze, Azienda Ospedaliera Integrata, Verona, Italy; ^6^Neurology Unit, Valduce General Hospital, Como, Italy; ^7^U.O. Neurologia, DAI Neuroscienze-Riabilitazione, Azienda Ospedaliera-Universitaria S. Anna, Ferrara, Italy; ^8^U.O. Neurologia, G. Jazzolino Hospital, Vibo Valentia, Italy; ^9^Istituto Neurologico Nazionale C. Mondino, Pavia, Italy; ^10^U.O.C. Stroke Unit, Dipartimento di Scienze Neurologiche e Neurosensoriali, Azienda Ospedaliera Universitaria Senese, Siena, Italy; ^11^Department of Neurosciences, Neurological Clinic, University of Pisa, Pisa, Italy; ^12^UOC di Neurologia e “Stroke Unit”, Ospedale San Bortolo, Vicenza, Italy; ^13^Neurology Unit, Arcispedale Santa Maria Nuova, Reggio Emilia, Italy; ^14^Department of Clinical Neurological Sciences, London Health Sciences Centre, Western University, London, ON, Canada; ^15^Neurology Unit, Misericordia e Dolce Hospital, Prato, Italy; ^16^Stroke Unit, Department of Neurology, Ospedale di Circolo e Fondazione Macchi, Varese, Italy; ^17^Department of Neurology, Istituti Ospitalieri, Cremona, Italy; ^18^Institute of Neuroscience, Italian National Research Council, Florence, Italy

**Keywords:** stroke, thrombolytic therapy, metalloproteinases, tissue inhibitor of metalloproteinases, stroke subtypes, hemorrhagic transformation, death

## Abstract

**Background:**

Experimentally, metalloproteinases (MMPs) play a detrimental role related to the severity of ischemic brain lesions. Both MMPs activity and function in tissues reflect the balance between MMPs and tissue inhibitors of metalloproteinases (TIMPs). We aimed to evaluate the role of MMPs/TIMPs balance in the setting of rtPA-treated stroke patients.

**Methods:**

Blood was taken before and 24-h after rtPA from 327 patients (mean age 68 years, median NIHSS 11) with acute ischemic stroke. Delta median values of each MMP/TIMP ratio [(post rtPA MMP/TIMP-baseline MMP/TIMP)/(baseline MMP/TIMP)] were analyzed related to symptomatic intracranial hemorrhage (sICH) according to NINDS criteria, relevant hemorrhagic transformation (HT) defined as confluent petechiae within the infarcted area or any parenchymal hemorrhage, stroke subtypes (according to Oxfordshire Community Stroke Project) and 3-month death. The net effect of each MMP/TIMP ratio was estimated by a logistic regression model including major clinical determinants of outcomes

**Results:**

Adjusting for major clinical determinants, only increase in MMP9/TIMP1 and MMP9/TIMP2 ratios remained significantly associated with sICH (odds ratio [95% confidence interval], 1.67 [1.17–2.38], *p* = 0.005; 1.74 [1.21–2.49], *p* = 0.003, respectively). Only relative increase in MMP9/TIMP1 ratio proved significantly associated with relevant HT (odds ratio [95% confidence interval], 1.74 [1.17–2.57], *p* = 0.006) with a trend toward significance for MMP9/TIMP2 ratio (*p* = 0.007).

**Discussion:**

Our data add substantial clinical evidence about the role of MMPs/TIMPs balance in rtPA-treated stroke patients. These results may serve to generate hypotheses on MMPs inhibitors to be administered together with rtPA in order to counteract its deleterious effect.

## Introduction

Matrix metalloproteinases (MMPs) are a family of zinc-dependent endopeptidases that are involved in extracellular matrix (ECM) degradation (1). The turnover of ECM is regulated by the balance between MMPs and a group of endogenous proteins called tissue inhibitor of metalloproteinases (TIMPs) (2). Active MMPs and some MMP proenzymes form 1:1 complexes with TIMPs and the unbalance between these two families of molecules appears implicated in a variety of diseases (3). A list of MMPs and TIMPs with their putative role in acute ischemic stroke is shown in Table S1 in Supplementary Material.

**Figure 1:**
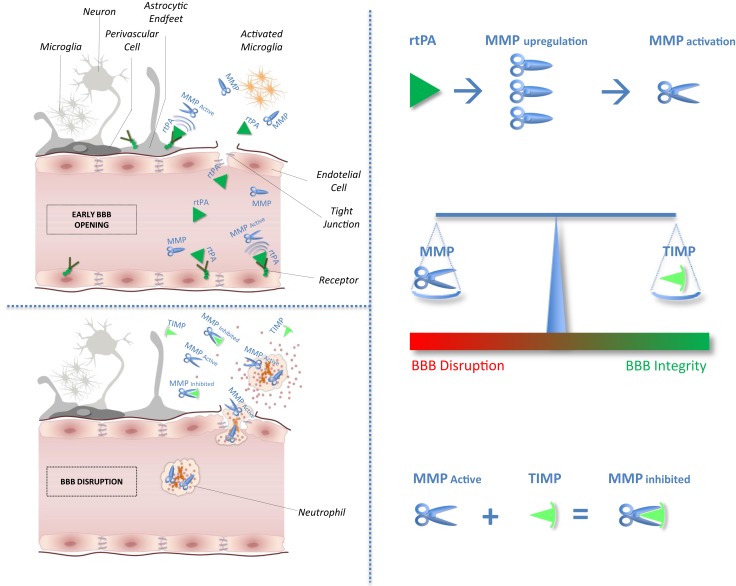
**Impact of tissue plasminogen activator on MMP/TIMP unbalance at the neurovascular unit level**. After acute ischemic stroke, rtPA may cross blood–brain barrier (BBB), enter the brain parenchyma, and thereby damage neurovascular unit components by promoting metalloproteinase (MMPs) production and activation. Indeed, unbalance between MMPs and their natural inhibitors (tissue inhibitors of metalloproteinases, TIMPs) may exacerbate BBB disruption leading to hemorrhagic transformation and edema of an ischemic brain lesion.

After cerebral ischemia, the general neuronal response to excitotoxic injury determines the release of pro-inflammatory cytokines that stimulate the local production of MMPs and TIMPs (4). In experimental models of brain ischemia, MMPs and MMP/TIMP unbalance play a detrimental role related to blood–brain barrier (BBB) disruption leading to hemorrhagic transformation and edema of an ischemic brain lesion (5). Circulating levels of MMP9 have been proved associated with poor outcomes in stroke patients treated with tissue plasminogen activator (rtPA) (6, 7). Furthermore, recent studies suggest that rtPA adverse effects may be mediated through MMPs upregulation and activation (2). No clinical study has hitherto considered selectively the effect of the balance between MMPs and their physiological inhibitor related to stroke outcomes after thrombolysis. Theoretical effects of rtPA on MMP/TIMP unbalance have been shown in Figure 1.

The aim of this study was to evaluate the effect of MMPs/TIMPs ratio on outcomes of ischemic stroke in the same cohort of the biological markers associated with acute ischemic stroke (MAGIC) study. Because MMP inhibition is considered a possible therapeutic target for stroke patients (8), a clearer understanding of MMP/TIMP interplay, compared with the effect of MMPs only, would have important implications for acute stroke therapies.

## Materials and Methods

### Study population

A detailed description of the biological MAGIC study is reported elsewhere (6). Patients were enrolled in 14 Italian centers and registered in the safe implementation of thrombolysis in stroke-international stroke thrombolysis register (SITS-ISTR), according to SITS-monitoring study criteria (9). The diagnosis of stroke was based on an expert’s clinical opinion and supported by neuroimaging. The Ethical Committee of the Careggi University Hospital in Florence approved the study protocol, and each center obtained ethical approval for data collection.

### Data collection

For each patient variables collected were

1. Baseline characteristics: age (years), sex (male), onset-to-treatment time (minutes), baseline NIHSS, blood glucose (mg/dl), and home medications.
2. Risk factors and comorbidities: history of hypertension, history of diabetes, history of hyperlipidemia, history of atrial fibrillation, history of congestive heart failure, and history of recent infections or inflammations.

Stroke severity was assessed using the NIHSS, administered before starting, at 24 h and 7 days after thrombolysis. Stroke types were categorized using the pathologically validated and largely used Oxfordshire Community Stroke Project (OCSP) classification (10), which distinguishes on syndromic basis four pictures: the total anterior circulation syndrome (TACS), the partial anterior circulation syndrome (PACS), the lacunar syndrome (LACS), and the posterior circulation syndrome (POCS). All patients underwent a CT scan at baseline to determine eligibility for treatment. In addition to the baseline CT scan before treatment, patients had a CT at 24 h, and at any time when clinical deterioration was observed.

The collection of the clinical d ata was blinded to the biomarkers’ results.

### Laboratory determinations

Blood samples were taken before and 24 h after rtPA. Blood was collected in tubes with anticoagulants (0.109M sodium citrate at ratio 9:1 or 1.8 mg/ml EDTA), as well as in tubes without anticoagulant, before starting and 24 h after thrombolysis. Tubes were centrifuged at room temperature at 1500 × *g* for 15 min, and the supernatants were stored in aliquots at −80°C until measurement of MMPs and TIMPs. Samples were analyzed in a unique central laboratory. Levels of different MMPs (MMP1, MMP2, MMP3, MMP7, MMP8, and MMP9) and TIMPs (TIMP1, TIMP2, and TIMP4) were determined using Bio-Plex suspension array system (Bio-Rad Laboratories Inc., Hercules, CA, USA) and R&D Kits (R&D System, Milan, Italy) following manufacturer’s instructions. The coefficient of variation of MMPs and TIMPs assays were 5.8 and 6.8%, respectively.

Biomarker measurement was blinded to clinical data. The relative pre- and post-thrombolysis variation of MMP/TIMP ratio [(post rtPA MMP/TIMP-baseline MMP/TIMP)/(baseline MMP/TIMP)] was considered as main explanatory variable.

### Outcomes

Delta median values of each MMP/TIMP ratio were analyzed related to

1. Symptomatic intracranial hemorrhage (sICH) defined as any neurologic deterioration occurring within 24 h after thrombolytic treatment and judged by the treating physician to be secondary to a new brain hemorrhage as shown by a head CT (11).
2. Relevant hemorrhagic transformation (HT) defined as hemorrhagic infarction type 2 and any type of parenchymal hemorrhage according to ECASS II criteria (12).
3. Subtypes of strokes defined according OCSP classification (10).
4. Death at 3 months.

### Statistical analysis

We used Pearson χ^2^ to test for significance while comparing categorical variables and ANOVA test for numeric variables. To analyze differences in biomarkers levels between baseline and 24 h, we choosed the non-parametric Mann–Whitney *U* test because of relatively large statistical variations. We considered a Bonferroni corrected *p*-value <0.007 to be statistically significant.

The net effect of the variation of each MMP/TIMP ratio on outcomes was then estimated by a logistic regression model, including as covariates age, sex, onset-to-treatment time, baseline blood glucose, baseline NIHSS, history of atrial fibrillation, history of congestive heart failure, statin use, aspirin use, antiplatelet use, and antihypertensive use. Since there were significant variations in the concentration of studied biomarkers across collaborating centers, in the multivariate analysis, we controlled also for center effect. History of inflammatory disorders or infections, occurred in the last 7 days, was entered as a potential modifier of MMPs or TIMPs variations.

We used Kruskal–Wallis test to study the bivariate association between delta values of MMPs or TIMPs and stroke subtypes (according to OCSP classification). Considering the small sample size of LACS and POCS subtypes in comparison with TACS, we used the dependent dichotomous variable, TACS vs. other syndromes, in the multivariate analysis. The independent association of MMP/TIMP ratio and ischemic stroke subtypes was analyzed with binary logistic regression models adjusting for age, sex, onset-to-treatment time, history of recent infection/inflammation, history of atrial fibrillation, history of hypertension, history of diabetes, history of congestive heart failure, statin use, aspirin use, other antiplatelet use, antihypertensive use, and center effect.

## Results

Between 2008 and 2011, 327 (mean age, 68.9 ± 12.1 years; 58% males) patients were enrolled in the study. A detailed description of the cohort enrollment has been reported in our previous paper (6). The rate of SICH according to the NINDS definition was 8.2%, relevant HT was present in 12.2% of the cohort, and mortality was 8.6%. The rate of TACS, the most severe ischemic stroke type, was 27.2%. Clinical and demographic characteristics of the 327 patients are shown in Table 1.

**Table 1:** **Baseline characteristic of the 327 enrolled patients**.

Baseline characteristics	All (*n* **=** 327)	No sICH (*n* **=** 300)	sICH(*n* **=** 27)	*p*-Value
Age, years, mean (SD)	68.9 (12.1)	68.6 (12.1)	72.2 (11.5)	0.142
Male, *n* (%)	190 (58.1)	175 (58.3)	15 (55.6)	0.779
Time OT, min, mean (SD)	163.5 (75.7)	164.3 (78.3)	154.8 (35.8)	0.534
NIHSS, median (IQR)	11 (7–16)	11 (7–16)	15 (9–20)	0.057
Glucose, mg/dl, mean (SD)	129.2 (47.9)	130.0 (48.7)	133.2 (38.8)	0.735
Hypertension, *n* (%)	197 (61.0)	181 (60.9)	16 (61.5)	0.952
Diabetes, *n* (%)	50 (15.4)	46 (15.5)	4 (14.8)	0.926
Hyperlipidemia, *n* (%)	81 (25.8)	74 (25.6)	7 (28.0)	0.793
Atrial fibrillation, *n* (%)	73 (22.7)	68 (23.1)	5 (18.5)	0.590
Congestive heart failure, *n* (%)	35 (10.9)	31 (10.5)	4 (15.4)	0.440
Aspirin	105 (32.4)	92 (31.0)	13 (48.1)	0.068
Other antiplatelets	36 (11.1)	32 (10.8)	4 (14.8)	0.522
Antihypertensives	168 (51.7)	152 (51.0)	16 (59.3)	0.411
Statins	31 (9.5)	29 (9.7)	2 (7.4)	0.701
Recent infection or inflammation, *n* (%)	43 (13.2)	41 (13.7)	2 (7.7)	0.388

*sICH, symptomatic intracerebral hemorrhage*.

*p calculated by Mann–Whitney U test for NIHSS, by ANOVA for other numeric variables, by Pearson χ^2^ for categorical variables.

### Pre- and post-thrombolysis variations of MMPs/TIMPs according to outcomes

**Figure 2:**
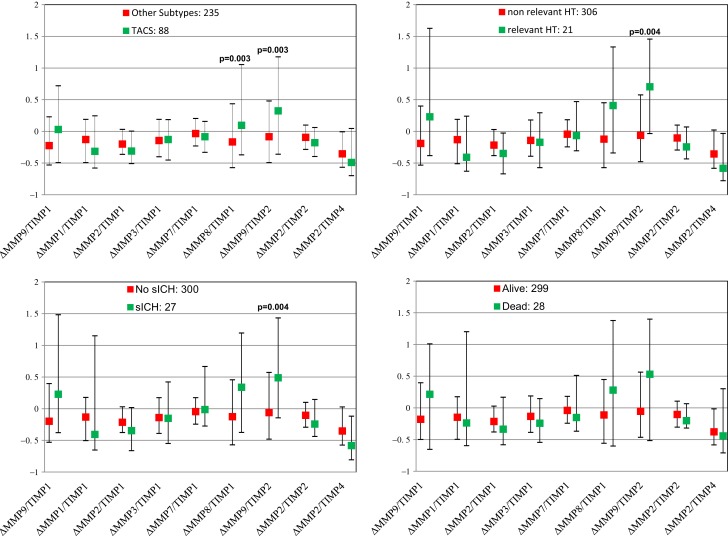
**Pre- and post-thrombolysis variations of matrix metalloproteinases (MMPs) and tissue inhibitors of metalloproteinases (TIMPs) ratio (**Δ=** [24 h MMP/TIMP **−** baseline MMP/TIMP]/baseline MMP/TIMP) in subgroups of patients with different unfavorable outcomes**. Values are shown as median and interquartile range. sICH indicates symptomatic intracerebral hemorrhage.

Figure 2 shows pre- and post-thrombolysis changes of each MMP/TIMP ratio in patients with and without sICH, in patients with and without relevant HT, in patients who died and in those who survived, and in patients with TACS compared with PACS. Table 2 shows multivariate analysis adjusted for major clinical confounders.

**Table 2:** **Effect of delta MMPs/TIMPs ratio on sICH, relevant HT, death, and TACS adjusting for major determinants**.

MMP/TIMP	sICH^a^ OR (95% CI)	*p*	Relevant HT^a^ OR (95% CI)	*p*	Death^a^ OR (95% CI)	*p*	TACS^b^ OR (95% CI)	*p*
MMP9/TIMP1	1.67 (1.17–2.38)	**0.005**	1.74 (1.17–2.57)	**0.006**	1.22 (0.92–1.63)	0.171	1.15 (0.93–1.42)	0.211
MMP1/TIMP1	1.01 (0.96–1.06)	0.704	1.01 (0.96–1.07)	0.615	0.97 (0.86–1.08)	0.554	1.02 (0.99–1.06)	0.260
MMP2/TIMP1	0.99 (0.81–1.21)	0.923	1.05 (0.86–1.29)	0.626	1.37 (1.00–1.87)	0.048	1.38 (1.06–1.80)	0.016
MMP3/TIMP1	0.85 (0.59–1.24)	0.404	0.88 (0.55–1.39)	0.579	1.00 (0.53–1.88)	0.991	1.06 (0.86–1.32)	0.587
MMP7/TIMP1	1.05 (0.97–1.13)	0.273	1.04 (0.96–1.13)	0.331	1.01 (0.92–1.11)	0.884	1.00 (0.94–1.07)	0.943
MMP8/TIMP1	0.99 (0.92–1.06)	0.788	1.00 (0.94–1.06)	0.889	1.00 (0.97–1.03)	0.730	1.01 (0.99–1.03)	0.585
MMP9/TIMP2	1.74 (1.21–2.49)	**0.003**	1.71 (1.16–2.52)	**0.007**	1.43 (1.02–1.99)	0.037	1.35 (1.03–1.78)	0.032
MMP2/TIMP2	0.99 (0.85–1.16)	0.912	1.04 (0.88–1.22)	0.652	1.27 (0.96–1.68)	0.098	1.38 (1.08–1.76)	0.009
MMP2/TIMP4	0.90 (0.68–1.19)	0.458	0.98 (0.75–1.27)	0.877	1.61 (1.10–2.36)	0.015	1.37 (1.05–1.77)	0.019

*Bold font indicates statistically significant data*.

*MMP, matrix metalloproteinase; TIMP, tissue inhibitors of metalloproteinase; OR, odds ratio; CI, confidence interval; sICH, symptomatic intracerebral hemorrhage; relevant HT, relevant hemorrhagic transformation; TACS, total anterior circulation syndrome*.

*^a^Binary logistic regression analysis adjustment for age, sex, onset-to-treatment time, baseline blood glucose, baseline NIHSS, history of atrial fibrillation, history of congestive heart failure, center effect, history of recent infection/inflammation, statin use, aspirin use, antiplatelet use, and antihypertensive use*.

*^b^Binary logistic regression analysis adjustment for age, sex, onset-to-treatment time, history of recent infection/inflammation, history of atrial fibrillation, history of hypertension, history of diabetes, history of congestive heart failure, statin use, aspirin use, other antiplatelet use, antihypertensive use, and center effect*.

At univariate analysis, only relative increase in MMP9/TIMP2 ratio proved significantly associated with sICH, while MMP9/TIMP1 ratio showed a trend toward significance (*p* = 0.004, *p* = 0.018, respectively). Similarly, only MMP9/TIMP2 ratio was significantly associated with relevant HT (*p* = 0.004).

Increase in MMP8/TIMP1 and MMP9/TIMP2 ratios proved significantly associated with TACS (*p* = 0.003, *p* = 0.003, respectively). None of the ratios examined was significantly associated with death. Adjusting for clinical determinants, among all ratios examined, only increase in MMP9/TIMP1 and MMP9/TIMP2 ratios remained significantly associated with sICH (*p* = 0.005, *p* = 0.003, respectively), whereas only relative increase in MMP9/TIMP1 ratio was significantly associated with relevant HT (*p* = 0.006) with a trend toward significance for MMP9/TIMP2 ratio (*p* = 0.007). A dose–response relationship was observed between the incidence of symptomatic intracranial hemorrhage and MMP9/TIMP1 ratio quartiles (4.9% of SICH in the first quartile; 6.1% in the second quartile, 7.4% in the third quartile, 14.6% in the fourth quartile, *p* = 0.025) and MMP9/TIMP2 ratio quartiles (4.9% of SICH in the first quartile; 3.7% in the second quartile, 8.5% in the third quartile, 15.9% in the fourth quartile, *p* = 0.006).

## Discussion

The primary observation of this study is that relative increase of MMP9/TIMP1 and MMP9/TIMP2 ratios was independently associated with sICH. In a previous study (6), we have already demonstrated that relative pre–post rtPA variation of just MMP-9 was associated with sICH. To examine whether, consistently with the biological rational, the effect of MMP9/TIMP 1–2 unbalance was stronger than MMP9 alone in determining sICH, we performed a stepwise regression analysis: only MMP9/TIMP1–2 ratios were selected as predictors of sICH when compared with MMP9 alone.

### Possible explanation

In experimental models of brain ischemia, tissue inhibitor of metalloproteinases has been shown to protect BBB, inhibiting MMP9 activity. Indeed, TIMP-1 knockout mice showed MMP9 overexpression and exacerbation of BBB leakage and ischemic injury (13).

The role of MMP/TIMPs unbalance has been poorly studied in human stroke setting. In 41 patients evaluated for acute stroke, MMP9 and MMP9/TIMP1 ratios were associated with BBB disruption visualized *in vivo* by FLAIR MRI (14).

An association between MMP9/TIMP1 ratio and the most severe ischemic stroke subtype was found in a cohort of 126 untreated stroke patients (15).

In a study examining human brain samples after a fatal stroke, MMP-9 and TIMP-2 demonstrated higher expression in brain microvessels, prompting the hypothesis of selectively targeting these molecules for “vasculoprotection” following stroke (16).

Concerning therapeutic strategies in stroke, a recent review of the literature discussed the current status of neuroprotection and extension of thrombolytic window by directly or indirectly inhibiting MMP-9 activity (8). These reports collectively indicate the importance of a balance between the levels of MMPs and their natural inhibitors TIMPs in maintaining the ECM integrity in ischemic stroke. However, the timing of inhibition is critical and late MMP9 inhibition may be deleterious, suggesting a role for MMP-9 in delayed cortical response and recovery after stroke (17).

### Strengths and limitations

The primary strength of this study is the relative large number of participants constituting the largest series hitherto investigated of rtPA-treated stroke patients in whom MMPs and TIMPs were measured before and after thrombolysis. A limitation of this study consists in the lack of a control group of patients not treated with thrombolysis. Furthermore, the use of activity assays to measure MMPs and TIMPs might improve the quality of future studies.

## Conclusion

Our data add substantial clinical evidence about the role of MMPs/TIMPs unbalance related to hemorrhagic transformation of an ischemic lesion after rtPA treatment. These results may serve to generate hypothesis on MMPs inhibitors to be administered early, possibly within the same time-window of rtPA therapy, in order to counteract its deleterious effect.

## Author Contributions

Study concept and design: BP, VP, MN, PN, BG, GP, DT, AN, RA, and DI. Acquisition of data: BP, VP, MN, PN, BG, GP, DT, AN, RA, DI, PT, EI, AS, ES, PB, MG, MT, DC, GM, RT, GO, FP, NM, FM, MD, GB, and MS. Statistical analysis: BP, VP, MN, PN, BG, GP, DT, AN, RA, and DI. Analysis and interpretation of data: BP, VP, MN, PN, BG, GP, DT, AN, RA, DI, PT, EI, AS, ES, PB, MG, MT, DC, GM, RT, GO, FP, NM, FM, MD, GB, and MS. Drafting and critical revision of manuscript: BP, VP, MN, PN, BG, GP, DT, AN, RA, and DI. Study supervision: BP, VP, MN, PN, BG, GP, DT, AN, RA, and DI.

## Conflict of Interest Statement

The authors declare that the research was conducted in the absence of any commercial or financial relationship that could construed as a potential conflicts of interest.

## Supplementary Material

The Supplementary Material for this article can be found online at http://www.frontiersin.org/article/10.3389/fneur.2015.00121/abstract
