# Supplementary material for: Unbalanced Metalloproteinase-9 and Tissue Inhibitors of Metalloproteinases Ratios Predict Hemorrhagic Transformation of Lesion in Ischemic Stroke Patients Treated with Thrombolysis: Results from the MAGIC Study
Source: Front Neurol. 2015 May 27;6:121. doi: 10.3389/fneur.2015.00121 (PMC4445323; doi:10.3389/fneur.2015.00121)
Supplement: Supplementary file 1 [file table_1.docx]

***Supplementary Material***

**Unbalanced Metalloproteinase-9 and Tissue inhibitors of Metalloproteinases ratio Predicts Hemorrhagic Transformation of Lesion in Ischemic Stroke Patients Treated with Thrombolysis: Results from the MAGIC Study**

Benedetta Piccardi, MD; Vanessa Palumbo, MD ; Mascia Nesi, MD; Patrizia Nencini, MD; Anna Maria Gori, BS; Betti Giusti, BS; Giovanni Pracucci, MD; Paolina Tonelli, MD; Eleonora Innocenti, MD; Alice Sereni BS; Elena Sticchi, BS; Danilo Toni, MD; Paolo Bovi, MD; Mario Guidotti, MD ; Maria Rosaria Tola, MD; Domenico Consoli, MD; Giuseppe Micieli, MD; Rossana Tassi, MD; Giovanni Orlandi, MD; Francesco Perini, MD , Norina Marcello, MD; Antonia Nucera, MD; Francesca Massaro, MD; DeLodovici Maria Luisa, MD; Giorgio Bono, MD; Maria Sessa, MD; Rosanna Abbate, MD; Domenico Inzitari, MD on behalf of the MAGIC Study Group.

**Supplementary Table 1.**

MMPs and TIMPs: denomination, function and associations with clinical outcomes in acute ischemic stroke patients treated with thrombolytic therapy

| **MMPs or TIMPS** | **DENOMINATION** | **SOURCES** | **MODULATORS** | **SUBSTRATES** | **ASSOCIATION WITH OUTCOMES** |
| --- | --- | --- | --- | --- | --- |
| MMP-2 | GELATINASE A | Astrocytes, endothelial cells, and potentially leukocytes | Endothelin-1, angiotensin II and interleukin-1 beta (IL-1Beta) | Intracellular alpha-actin, desmin,GSK-3Beta, TnI, Titin, MLC. Extracellular Collagen type IV and tight junction proteins. Activates CXCL8, Tumor Necrosis Alpha (TNF-alpha) | MMP-2 levels did not differ in terms of presence or absence of haemorrhagic transformation considered globally (Montaner J 2001) |
| MMP-9 | GELATINASE B | Astrocytes, neurons, Microglial cells, vascular cells, astrocytes, neuronal cells and leukocytes | Inflammatory markers, reactive oxygen species  proMMP-9 was activated by plasmin | Degrades: Collagen type I, II, III, IV, V, XI, XVI, fibronectin, laminin, osteopontin, thrompospondin-1, tenascin-C, galectin-3, decorin  Activates: CXCL5, CXCL8, TNF-alpha, IL-1 Beta, TGF-Beta  Inactivates: CXCL1, CXCL4, CXCL5, CXCL7, CXCL12, IL-1 Beta | MMP-9 level predicts late haemorrhagic infarction and a 24-hour peak precedes early parenchymal hematoma (Montaner J 2001).  Baseline MMP-9 level predicts partial haemorrhage appearance after t-PA treatment (Montaner J 2003).  Hyperacute plasma MMP9 was higher in stroke patients treated with tPA compared with tPA-untreated stroke patients and control subjects without stroke (Ning et al., 2006). |
| MMP-3 | Stromelysin-1 | Monocytes, microglial cells and pericytes. | Cytokines, reactive oxygen species (ROS), growth factors and cell–cell/cell–ECM interactions  Both intra- and extracellular activities of MMP-3 can be regulated by TIMPs, among which the TIMP-1/MMP-3 ratio is the best characterized  t-PA induces stromelysin-1 (MMP-3) in endothelial cells through activation of lipoprotein receptor-related protein.  proMMP-3 was activated by plasmin | Activates growth factors and the zymogen forms of pro-MMP-1, -3, -7, -8, -9 and -13  Cleaves cell adhesion molecules, chemokines, cytokines.  Degrades proteoglycans, fibronectin, fibrillin, laminin and elastin | MMP3 gene polymorphisms were associated with ischemic stroke, but not with intracerebral haemorrhage in the Korean population (Kim et al., 2012) |
| MMP-1 | COLLAGENASE-1 | Astrocytes, pericytes, chondrocytes, dendritic cells, endothelial cells, fibroblasts, macrophages, and leukocytes | proMMP-1 was activated by plasmin | Degrades fibrillar collagens of types I, II, III, V, and XI | None |
| MMP-8 | COLLAGENASE-2 | Neutrophil degranulation  Endothelial cells, smooth muscle cells and macrophages within human atherosclerotic lesions | ROS released from activated neutrophils. Proteases like cathepsin G, chymotrypsin, or MMPs (-3, -7, -10, and -14)  Inflammatory cytokines (such as IL-β or CD40L | Degrades elastin, type I and IV collagen, fibronectin, vitronectin, aggrecan, proteoglycans, serum IGFBP-1, -2, -3, -4, -5, -6 and plasminogen | None |
| MMP-7 | MATRILYSIN 1 | Endothelial cells, monocytes, macrophages | MMP-3 activates MMP7.  proMMP-7 was activated by plasmin | Degrades collagens IV and X, gelatin, casein, laminin, aggrecan, entactin, elastin, versican, and fibrinogen  Processes cell surface molecules such as pro-alpha-defensin, Fas-ligand, tumor necrosis factor-alpha, and E-cadherin. | None |
| TIMP-1 | TISSUE INHIBITOR OF METALLO-PROTEINASE-1 |  | Inflammatory markers such as CRP, IL-1Beta and TNF-alpha.  Early rise (2–6 h after symptom onset )of TIMP-1 was related to the early increase in MMP-9, the prolongation of increased TIMP-1 levels in patients with severe stroke may be due to processes of post-ischemic recovery, as TIMP-1.  Increasing doses of tPA are associated with increasing TIMP-1 levels in the cortex and the basal ganglia | All TIMPs are capable of inhibiting all known MMPs; however, the efficacy of MMP inhibition varies with each TIMP (Arpino et al., 2015)  TIMP-2 and TIMP-1 preferentially inhibit MMP-2 and MMP-9, respectively. | In acute ischemic stroke treated with thrombolysis ,TIMP -1 variation after thrombolysis Is associated with hemorrhagic transformation of lesion and death (Inzitari et al., 2013). |
| TIMP-2 | TISSUE INHIBITOR OF METALLO-PROTEINASE-2 |  | Increasing doses of tPA are associated with increasing TIMP-2 levels in the cortex and the basal ganglia | All TIMPs are capable of inhibiting all known MMPs; however, the efficacy of MMP inhibition varies with each TIMP (Arpino et al., 2015).  TIMP-2 and TIMP-1 preferentially inhibit MMP-2 and MMP-9, respectively, Although TIMP-2 is an inhibitor of MMPs, paradoxically it can function as a co-activator of proMMP-2 depending upon the concentration of TIMP-2 protein. TIMP-2 may act on MT1-MMP | None |
| TIMP-4 | TISSUE INHIBITOR OF METALLO-PROTEINASE-4 |  | When TIMP-4 is coexpressed with TIMP-2, it inhibits the activation of latent MMP-2 via MMP-14 . | MMP-1, MMP-2, MMP-3, MMP-7, and MMP-9 | Variations of TIMP1 levels were significantly associated with death (Inzitari et al., 2013). |

References

Lakhan SE, Kirchgessner A, Tepper D, Leonard A. Matrix Metalloproteinases and Blood-Brain Barrier Disruption in Acute Ischemic Stroke. *Front Neurol.* (2013) 4: 32. doi: 10.3389/fneur.2013.00032

Montaner J, Alvarez-Sabín J, Molina CA, Anglés A, Abilleira S, Arenillas J, Monasterio J. Matrix metalloproteinase expression is related to hemorrhagic transformation after cardioembolic stroke. *Stroke*. 2001 Dec 1;32(12):2762-7.

Montaner J, Molina CA, Monasterio J, Abilleira S, Arenillas JF, Ribó M, Quintana M, Alvarez-Sabín J. Matrix metalloproteinase-9 pretreatment level predicts intracranial hemorrhagic complications after thrombolysis in human stroke. *Circulation.* 2003 Feb 4;107(4):598-603.

Ning M, Furie KL, Koroshetz WJ, Lee H, Barron M, Lederer M, Wang X, Zhu M, Sorensen AG, Lo EH, Kelly PJ. Association between tPA therapy and raised early matrix metalloproteinase-9 in acute stroke. *Neurology.* 2006 May 23;66(10):1550-5.

Inzitari D, Giusti B, Nencini P, Gori AM, Nesi M, Palumbo V, Piccardi B, Armillis A, Pracucci G, Bono G, Bovi P, Consoli D, Guidotti M, Nucera A, Massaro F, Micieli G, Orlandi G, Perini F, Tassi R, Tola MR, Sessa M, Toni D, Abbate R; MAGIC Study Group. MMP9 variation after thrombolysis is associated with hemorrhagic transformation of lesion and death. *Stroke.* 2013 Oct;44(10):2901-3.

Expression of TIMPs has been found in several types of cells and
tissues, see for details:
[http://www.genecards.org/cgi-bin/carddisp.pl?gene=TIMP4&search=82be527ade0e8dad1a8e2c2dfe3b1034](http://www.genecards.org/cgi-bin/carddisp.pl?gene=TIMP4&search=82be527ade0e8dad1a8e2c2dfe3b1034)

[http://www.genecards.org/cgi-bin/carddisp.pl?gene=TIMP2&search=5f1718211069cbe1dd5e4f0ecab57153](http://www.genecards.org/cgi-bin/carddisp.pl?gene=TIMP2&search=5f1718211069cbe1dd5e4f0ecab57153)

[http://www.genecards.org/cgi-bin/carddisp.pl?gene=TIMP1&search=960481fe1cb24a55343cd454322685cb](http://www.genecards.org/cgi-bin/carddisp.pl?gene=TIMP1&search=960481fe1cb24a55343cd454322685cb)
